# Genome Analysis of 17 Extensively Drug-Resistant Strains Reveals New Potential Mutations for Resistance

**DOI:** 10.1128/genomeA.00759-14

**Published:** 2014-07-31

**Authors:** H. Guio, D. Tarazona, M. Galarza, V. Borda, R. Curitomay

**Affiliations:** a Laboratorio de Biotecnología y Biología Molecular, Instituto Nacional de Salud (INS), Lima, Peru; b Departamento de Neumología, Hospital Nacional Hipólito Unanue, Lima, Peru; c Asociación Latinoamericana de Biotecnología (NGO-ALBIOTEC), Lima, Peru

## Abstract

We report the whole-genome sequence of an extensively drug-resistant (XDR) tuberculosis (TB) strain of Latin American–Mediterranean (LAM) lineage. This strain is phenotypically resistant to aminoglycosides, but carries no related mutations in *rrs*, *tlyA*, and *eis*. Through genome analysis comparison with 16 XDR strains, we found 218 non-synonymous single nucleotide polymorphisms (SNPs) shared that could confer resistance.

## GENOME ANNOUNCEMENT

For Peru, the incidence rate for tuberculosis (TB) in 2012 was 95/100,000 population, from which 0.3% were new cases of extensively drug-resistant (XDR) TB. 94% of these cases were from Lima (1). XDR-TB strains are resistant to isoniazid (INH), rifampicin (RIF), fluoroquinolones (FLQ), and at least one of the three injectable anti-TB drugs: amikacin (AMK), capreomycin (CAP), or kanamycin (KAN) (2). In 1999, Peru reported one case with this resistant pattern. However, the concept of XDR-TB appears in 2006 with the first outbreak in KwaZulu-Natal (KZN), South Africa (3).

In our quest to identify new possible mutations that can confer resistance in XDR-TB strains, we analyzed the XDR strain, INS-XDR, isolated from a Peruvian patient with active tuberculosis. We determined the strain’s lineage to be Latin American–Mediterranean (LAM) using 24 mycobacterial interspersed repetitive unit-variable number of tandem repeat (MIRU-VNTR) loci (4) and SpolPred (5). The genomic DNA was sequenced on an Illumina HiSeq 2000 with 1,140× coverage. Sequence reads for the isolates were mapped against the corrected reference genome *M. tuberculosis* strain H37Rv The average coverage was 82 for INS-XDR (49,793,402 mapped reads) and assembly with BWA v0.5.9-r16 (6), using H37Rv genome (AL123456.3) (7) as a reference. For annotation process, we used Rapid Annotations using Subsystem Technology (8) and the Prokaryotic Genome Annotation Pipeline. The sequencing data generated has 49,793,402 paired-ends reads. The assembling process resulted in 20 contigs that represents 99.98% of the H37Rv genome. The INS-XDR has a total of 4,391,020 pb with an average G+C content of 65.4%. We used Sanger sequencing to confirm specific mutations. Phenotypically, this strain was resistant to RIF, INH, FLQ, KAN, and CAP. However, sequence analysis of *rrs* and the promoter *eis* do not show mutations for aminoglycosides resistance (9).

To analyze possible mutations causing resistance to CAP and KAN, a polymorphism study was carried out by comparing INS-XDR against KZN605 (a XDR-TB strain of LAM linage) and 16 reported TB-XDR genomes from China, FJ05194, GuangZ0019 (10), XDR1219, XDR1221 (11), GuangZ0008, 293, FJ07070, GuangZ0017, GuangZ0026 (12); Peru, Peru_04_R0292, Peru_03_R0268 (13); Russia, CTRI-4 (14), Russia_03-R1082 (13); Malaysia, UM1072388579 (15), and South Africa, KZN_R506 (16) and KZN_605. Single nucleotide polymorphisms (SNPs) were identified using SNPsFinder (17) and SNP tree (18), and were classified in the Clusters of Orthologous Groups (COG) database (19). These analyses showed evidence of 218 non-synonymous SNPs mainly in four COGs: (1) replication, recombination and repair, L, *n*=14, (2) energy production and conversion, C, *n*=14, (3) amino acid transport and metabolism, E, *n*=12, and (4) secondary metabolites biosynthesis, transport, and catabolism, Q, *n*=11. These 218 non-synonymous SNPs belong to 125 genes. Given that CAP and KAN act by inhibiting protein synthesis and causing production of abnormal proteins, we hypothesize that the 14 non-synonymous SNPs belonging to the C category would be the principal SNPs responsible for CAP and KAN resistance. This provides evidence that it may be worthwhile to explore these new SNPs for diagnosis of XDR-TB.

### Nucleotide sequence accession numbers.

This whole-genome shotgun project has been deposited at DDBJ/EMBL/GenBank under the accession JANH00000000. The version described in this paper is version JANH01000000.

## References

[1] ESNPyCTB 2012 Informe de la Estrategia Sanitaria Nacional de Prevención y Control de la Tuberculosis sobre Situación y Control de TB-MDR y TB-XDR en Perú. Estrategia Sanitaria Nacional de Prevención y Control de la Tuberculosis

[2] Jassal M Bishai WR 2009 Extensively drug-resistant tuberculosis. Lancet Infect. Dis. 9:19–30. 10.1016/S1473-3099(08)70260-3 18990610

[3] Gandhi NR Moll A Sturm AW Pawinski R Govender T Lalloo U Zeller K Andrews J Friedland G 2006 Extensively drug-resistant tuberculosis as a cause of death in patients co-infected with tuberculosis and HIV in a rural area of South Africa. Lancet 368:1575–1580. 10.1016/S0140-6736(06)69573-1 17084757

[4] Supply P Allix C Lesjean S Cardoso-Oelemann M Rüsch-Gerdes S Willery E Savine E de Haas P van Deutekom H Roring S Bifani P Kurepina N Kreiswirth B Sola C Rastogi N Vatin V Gutierrez MC Fauville M Niemann S Skuce R Kremer K Locht C van Soolingen D 2006 Proposal for standardization of optimized mycobacterial interspersed repetitive unit-variable-number tandem repeat typing of *Mycobacterium tuberculosis*. J. Clin. Microbiol. 44:4498–4510. 10.1128/JCM.01392-06 17005759 PMC1698431

[5] Coll F Mallard K Preston MD Bentley S Parkhill J McNerney R Martin N Clark TG 2012 SpolPred: Rapid and accurate prediction of *Mycobacterium tuberculosis* spoligotypes from short genomic sequences. Bioinformatics 28:2991–2993. 10.1093/bioinformatics/bts544 23014632 PMC3496340

[6] Li H Durbin R 2009 Fast and accurate short read alignment with Burrows-Wheeler transform. Bioinformatics 25:1754–1760. 10.1093/bioinformatics/btp324 19451168 PMC2705234

[7] Comas I Coscolla M Luo T Borrell S Holt KE Kato-Maeda M Parkhill J Malla B Berg S Thwaites G Yeboah-Manu D Bothamley G Mei J Wei L Bentley S Harris SR Niemann S Diel R Aseffa A Gao Q Young D Gagneux S 2013 Out-of-Africa migration and *Neolithic coexpansion* of *Mycobacterium tuberculosis* with modern humans. Nat. Genet. 45:1176–1182. 10.1038/ng.2744 23995134 PMC3800747

[8] Overbeek R Olson R Pusch GD Olsen GJ Davis JJ Disz T Edwards RA Gerdes S Parrello B Shukla M Vonstein V Wattam AR Xia F Stevens R 2014 The SEED and the Rapid Annotation of microbial genomes using Subsystems Technology (RAST). Nucleic Acids Res. 42:D206–D214. 10.1093/nar/gkt1226 24293654 PMC3965101

[9] Sandgren A Strong M Muthukrishnan P Weiner BK Church GM Murray MB 2009 Tuberculosis drug resistance mutation database. PLOS Med. 6:e2. 10.1371/journal.pmed.1000002 19209951 PMC2637921

[10] Lin N Liu Z Zhou J Wang S Fleming J 22 8 2013 Draft genome sequences of two super-XDR isolates of *Mycobacterium tuberculosis* from China. FEMS Microbiol. Lett. 10.1111/1574-6968.12238 23965132

[11] Wu W Zheng H Zhang L Wen Z Zhang S Pei H Yu G Zhu Y Cui Z Hu Z Wang H Li Y 2013 *A genome-wide* analysis of multidrug-resistant and extensively drug-resistant strains of *Mycobacterium tuberculosis* Beijing genotype. Mol. Genet. Genomics 288:425–436. 10.1007/s00438-013-0758-4 23801408

[12] Zhang H Li D Zhao L Fleming J Lin N Wang T Liu Z Li C Galwey N Deng J Zhou Y Zhu Y Gao Y Wang T Wang S Huang Y Wang M Zhong Q Zhou L Chen T Zhou J Yang R Zhu G Hang H Zhang J Li F Wan K Wang J Zhang X-E Bi L 2013 Genome sequencing of 161 *Mycobacterium tuberculosis* isolates from China identifies genes and intergenic regions associated with drug resistance. Nat. Genet. 45:1255–1260. 10.1038/ng.2735 23995137

[13] Farhat MR Shapiro BJ Kieser KJ Sultana R Jacobson KR Victor TC Warren RM Streicher EM Calver A Sloutsky A Kaur D Posey JE Plikaytis B Oggioni MR Gardy JL Johnston JC Rodrigues M Tang PK Kato-Maeda M Borowsky ML Muddukrishna B Kreiswirth BN Kurepina N Galagan J Gagneux S Birren B Rubin EJ Lander ES Sabeti PC Murray M 2013 Genomic analysis identifies targets of convergent positive selection in drug-resistant *Mycobacterium tuberculosis*. Nat. Genet. 45:1183–1189. 10.1038/ng.2747 23995135 PMC3887553

[14] Ilina EN Shitikov EA Ikryannikova LN Alekseev DG Kamashev DE Malakhova MV Parfenova TV Afanas’ev MV Ischenko DS Bazaleev NA Smirnova TG Larionova EE Chernousova LN Beletsky AV Mardanov AV Ravin NV Skryabin KG Govorun VM 2013 Comparative genomic analysis of *Mycobacterium tuberculosis* drug resistant strains from Russia. PLoS One 8:e56577. 10.1371/journal.pone.0056577 23437175 PMC3577857

[15] Ng KP Yew SM Chan CL Chong J Tang SN Soo-Hoo TS Na SL Hassan H Ngeow YF Hoh CC Lee KW Yee WY 2013 Draft Genome Sequence of the first Isolate of Extensively drug-Resistant (XDR) *Mycobacterium tuberculosis* in Malaysia. Genome Announc. 1(1):e00056-12. 10.1128/genomeA.00056-12 23405310 PMC3569299

[16] Ioerger TR Koo S No E-G Chen X Larsen MH Jacobs WR Jr Pillay M Sturm AW Sacchettini JC 2009 Genome analysis of multi- and extensively-drug-resistant tuberculosis from KwaZulu-Natal, South Africa. PLoS One 4:e7778. 10.1371/journal.pone.0007778 19890396 PMC2767505

[17] Song J Xu Y White S Miller KW Wolinsky M 2005 SNPsFinder—a web-based application for genome-wide discovery of single nucleotide polymorphisms in microbial genomes. Bioinformatics 21:2083–2084. 10.1093/bioinformatics/bti176 15691853

[18] Leekitcharoenphon P Kaas RS Thomsen MC Friis C Rasmussen S Aarestrup FM 2012 snpTree—a web-server to identify and construct SNP trees from whole genome sequence data. BMC Genomics 13(Suppl 7):S6. 10.1186/1471-2164-13-S7-S6 23281601 PMC3521233

[19] Tatusov RL Fedorova ND Jackson JD Jacobs AR Kiryutin B Koonin EV Krylov DM Mazumder R Mekhedov SL Nikolskaya AN Rao BS Smirnov S Sverdlov AV Vasudevan S Wolf YI Yin JJ Natale DA 2003 The COG database: an updated version includes eukaryotes. BMC Bioinformatics 4:41. 10.1186/1471-2105-4-41 12969510 PMC222959

